# Chemotherapy response evaluation in metastatic non-small cell lung carcinoma (NSCLC) with 18F- FDG PET/CT and CT scan

**DOI:** 10.1186/1470-7330-14-S1-P10

**Published:** 2014-10-09

**Authors:** F Fathinul, N Abdul Jalil

**Affiliations:** 1 Centre for Diagnostic Nuclear imaging, University Putra Malaysia, 43400 UPM Serdang, Selangor Malaysia

## Background

Therapeutic response assessment in NSCLC is fundamental in order to avert futile surgery and an ineffective toxic chemotherapy. This study sought to determine the potential of the PET response criteria over the RECIST Criteria 1.1 in evaluating the treatment response of the NSCLC patients.

## Materials and methods

Eighteen patients (age; 60.16±5.39 years) with metastatic NSCLC were evaluated after 4 courses of chemotherapy and/or target therapy. [18F]-fluorodeoxyglucose with positron-emission tomography (PET) and computed tomography (CT) scans were subsequently used to assess tumour response. Discrepancies between the two procedures were evaluated after 4 courses of chemotherapy of the primary tumour lesion using the Response Evaluation Criteria in Solid Tumours (RECIST 1.1) and European Organisation for Research and Treatment of Cancer (EORTC) criteria evaluating CT and PET respectively.

## Results

Five morphologically partial responses (PR), two morphologically stable disease (SD), one morphologically progressive disease (PD) and two morphologically complete morphological response (CR) were correlated with the PET metabolic response criteria respectively. Two (PR) were evaluated as 2 complete metabolic response (CMR). Three cases of (SD) were evaluated as 3 CMR. Three morphologically (PD) were evaluated as 1 CR, 1 PMR and 1 SMD. There were significant difference between the SUVmax changes; mean: 0.51± 0.29 g/ml versus size changes; mean 0.30± 0.24 cm, p < 0.05. Conclusion: The RECIST 1.1 and the EORTC are two discordant methods in evaluating the therapeutic response of the metastatic NSCLC with the potential of greater parametric changes of the SUV in alluding changes of the tumour metabolic landscape.

